# Are there mappable genes for family resemblance for the magnitude of intra-individual variation in systolic blood pressure?

**DOI:** 10.1186/1471-2156-4-S1-S11

**Published:** 2003-12-31

**Authors:** Jennifer Lin, Anthony Hinrichs, Brian K Suarez

**Affiliations:** 1 Division of Preventive Medicine, Brigham and Women's Hospital, Harvard Medical School, Boston, Massachusetts, USA; 2 Department of Psychiatry, Washington University School of Medicine, St. Louis, Missouri, USA; 3 Department of Genetics, Washington University School of Medicine, St. Louis, Missouri, USA

## Abstract

**Background:**

The genetic regulation of variation in intra-individual fluctuations in systolic blood pressure over time is poorly understood. Analysis of the magnitude of the average fluctuation of a person's systolic blood pressure around his or her age-adjusted trend line, however, shows moderate, albeit significant, family resemblance in Cohort 1 of the Framingham Heart Study. To determine whether genomic regions affecting this phenotype could be identified, we pursued a "model-free" multipoint quantitative linkage analysis.

**Results:**

Two different linkage methods revealed multiple nominally significant signals, two to four of which are "replicated" in Cohort 2. When both cohorts are assembled into extended pedigrees, three linkage signals remain nominally significant by one or both methods.

**Conclusion:**

Any or all of the genomic regions in the vicinity of D5S1456, D11S2359, and D20S470 may contain elements that regulate systolic blood pressure homeostasis.

## Background

We used the rich longitudinal Framingham Heart Study data to explore the hypothesis that there is a heritable component to intra-individual variation in systolic blood pressure (SBP). Our strategy was to: 1) document family resemblance for the variability of SBP in Cohort 1; 2) use multipoint quantitative linkage analysis to identify genomic regions that may harbor genes that affect within-person variability in SBP; and 3) replicate any findings in Cohort 2. Despite the inherent differences between the two cohorts, if one or more linkage signals are replicated, we will combine the cohorts and repeat the analysis on the extended pedigrees.

We are particularly interested in assessing variation in the context of homeostatic regulation. It has been shown that a locus influencing age-adjusted SBP maps to chromosome 17q in these data [1]. We hypothesize that there is, additionally, familial resemblance for the magnitude of the fluctuations about each individual's age-related mean SBP. Since we anticipate that different individuals may have unique age-related trends (e.g., increasing SBP with age in some individuals, declining SBP in other individuals) and since we are not interested in the age-related means, *per se*, but rather the size of the fluctuations, we defined the phenotype as the average of absolute residual (AVGRES) obtained by regressing each person's SBP measurements on his or her age when the measurements were obtained. Because we wanted to be assured of having a sufficient number of measurements per person, we restricted our Cohort 1 analysis to subjects whose SBP was measured on a minimum of ten occasions. The mean number of SBP measurements for the subsets of Cohort 1 was 19 (SD = 3.5). See Dawber [2] for a description of design of the Framingham Heart Study.

## Methods

### Familial resemblance in Cohort 1

First we evaluated the distribution of AVGRES and found it to be significantly skewed and leptokurtotic in both males and females. A logarithmic (base 10) transformation rendered AVGRES normally distributed in both genders (for males, *p *= 0.35, and for females, *p *= 0.46). All subsequent analyses were carried out on the transformed values of AVGRES.

To assess family resemblance for AVGRES we used the FCOR program from the S.A.G.E. computer package [3]. Table 1 reports the gender-specific sib-pair correlations and their respective sample sizes. Modest, albeit significant, correlations in the range of 0.2 to 0.3 were obtained, and all are substantially higher than the spousal correlation of 0.045.

### Linkage analysis

Our plan was to analyze all available sib pairs from Cohort 1 for linkage with the objective of developing hypotheses that could be tested in Cohort 2. Unfortunately, only a small subset of the Cohort 1 subjects (that were used to assess family resemblance) were genotyped. The distribution of genotyped sibs is as follows: 38 pairs, 6 trios, and 1 quintet.

To perform multipoint quantitative linkage analysis for log AVGRES, we chose two methods implemented in the GENEHUNTER linkage program: nonparametric (NP) and expectation maximization Haseman-Elston (EM-HE). The NP method performs a Wilcoxon rank-sum test by first summing the ranks of absolute trait difference from sib pairs, multiplied by a simple weight based on the number of alleles shared identically by descent (IBD). Z scores are then obtained by the usual method [4].

The EM-HE procedure is based on the traditional Haseman-Elston method of regressing the squared sib-pair trait difference on the proportion of alleles shared IBD. In addition, when genotype information is missing, the EM algorithm infers the probability of alleles shared IBD by taking into account the allele sharing distribution as well as the regression parameters estimated from the real data points [5]. Similar to the NP score, the EM-HE test statistic for the regression coefficient is asymptotically normally distributed with mean 0 and unit variance. The GENEHUNTER 'pairs used' option was set to 3, which corresponds to 'all pairs of affected/phenotyped sibs.' This option weighs a sibship's contribution according to the number of independent pairs it contains, and guards against the possibility of large sibships dominating the results [6-9].

## Results

### Cohort 1

Table 2 reports all markers that attained nominal significance at the 5% level (i.e., score ≥ 1.645). Linkage signals at four markers were detected with just the NP statistic, 25 with just the EM-HE statistic; 11 signals were detected with both statistics.

### Cohort 2

The members of Cohort 2 have a maximum of only five SBP assessments (taken over a period of about 20 years). We felt that performing the regression analysis on subjects with fewer than five measurements could not adequately capture the sort of variation we are interested in. Indeed, the relative paucity of Cohort 2 measures may render it fundamentally different from Cohort 1. Restricting the analysis to genotyped sibs who were older than age 20 and had all five SBP assessments, however, yielded a much larger sample size compared to Cohort 1: 151 pairs, 84 trios, 35 quartets, 7 quintets, 4 sextets, and 2 septets.

Table 2 reports the NP and EM-HE linkage scores for those markers that attained nominal significance in Cohort 1. As expected, few of the original signals could be validated. If we count as a "replication," markers with a *p*-value < 0.05 obtained under the same weighting and statistical methodology, then there are two replications: D5S1456 for the NP approach and D11S2359 for the EM-HE approach. Two additional markers (D1S1653 and D20S470) revealed a "reversal". Both of these markers gave a nominally significant signal in Cohort 1 with the EM-HE statistic, but in Cohort 2 it was the NP statistic that achieved nominal "replication".

### Extended pedigrees

Combining available individuals from both cohorts results in 17 new families that were not analyzed in either cohort. Specifically, there are two half-sib pair families, two sib-pair families (that straddle the cohorts), and 13 families with additional avuncular or cousin members. Five large pedigrees were disassembled at a marriage node into smaller units. A total of 305 families were used for the combined analyses.

Table 3 reports the results of the multipoint linkage analysis in the extended pedigrees for the four "replicated" markers (and their closest neighbors). Compared to the NP and EM-HE scores obtained from the sib-pair analysis of Cohort 2, the scores for the extended families reflect some degradation, although all but D1S1653 remain nominally significant by one or the other method.

## Discussion

Physiological homeostasis is the process whereby a narrow range of phenotypes develops in the presence of wide variation in genotypes and environments. A classic example is the ability of homeothermic mammals to maintain a constant body temperature despite substantial genetic variation and fluctuating ambient air temperatures. The concept of physiological homeostasis bears some similarity to Waddington's notion of "canalization" [10] although this latter concept is usually applied to growth and developmental homeostasis.

That a breakdown in physiological homeostasis can result in either an increase or decrease in a phenotype — thus increasing the variance — is recognized in the operational definitions of many disorders. For example, among the *Diagnostic and Statistical Manual of Mental Disorders *(4th ed.) criteria for major depression are the following: a significant decrease or increase in appetite, insomnia or hypersomnia, psychomotor agitation or psychomotor retardation.

While the preponderance of genetic studies concentrate on the first moment of various phenotypes — and for medical reasons are usually interested in understanding deviantly high (or, rarely, deviantly low) phenotypes — it is reasonable to suppose that the magnitude of an individual's phenotypic variation is to some extent under genetic control [11]. Indeed, recently it has been shown that with respect to short-term (24-hour) variation among hypertensive patients, variability in blood pressure is positively related to organ damage and cardiovascular morbidity [12].

We used the rich longitudinal Framingham Heart Study data to study the genetics of long term variation in SBP. Our findings indicate that there is moderate familial resemblance for the magnitude of the deviation of a person's SBP from his or her unique age-related trend line. At least three caveats need to be mentioned. First, we restricted our Cohort 1 analysis to individuals with 10 or more measurements. Because this cohort was relatively small, we chose to include all subjects who met the above criterion, regardless of whether they were under treatment for hypertension. Subsequent to the Genetic Analysis Workshop 13, however, we carried out an analysis to determine if there exists a relationship between medication usage and AVGRES. We quantified medication usage as the proportion of SBP assessments where the subject was reported to be medicated. Unexpectedly, there is a strong positive correlation for both cohort 1 (*r *= 0.51) and Cohort 2 (*r *= 0.29) in the Framingham Heart Study. This previously unrecognized relationship between the use of hypertension medication and AVGRES is likely to have confounded our linkage analysis in an unknown fashion. Second, we did not adjust the data for body mass index (BMI)-a covariate known to show familial resemblance and to affect SBP. Since our phenotype was defined as the average residual from each subject's unique regression line it is unclear what, if any, effect the failure to adjust for BMI had on our results. Third, we did not test whether curvilinear regression would have provided an improved fit compared to simple linear regression. This decision was based on two considerations. First, if for some of the subjects, a quadratic or cubic function were found to fit significantly better than the linear component, its use in a subset of the data would have created a heterogeneous definition of the phenotype. Second, we were aware that the maximum number of available SBP measures on the Cohort 2 subjects was five, and it seemed frivolous to fit a high order function to such meager data.

**Figure 1:**
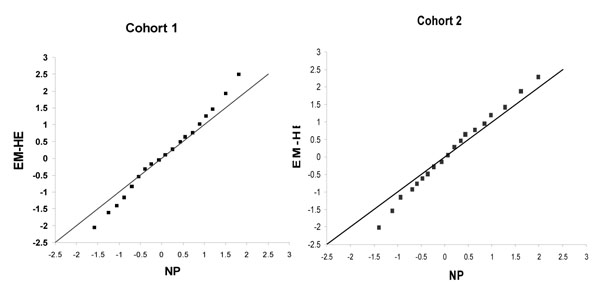
Quantile-quantile plots in 5% increments of the linkage statistics for Cohort 1 and Cohort 2

For the linkage analysis, we chose to analyze the sibship data with two different methods. Although the NP and the EM-HE methods are similar, as are all linkage methods, they are not identical. For the data we analyzed, the correlation between the NP and EM-HE scores were 0.72 and 0.69 for Cohorts 1 and 2, respectively. Inspection of the quantile-quantile (QQ) plots (Fig. 1) reveals that the NP statistic is more conservative than the EM-HE statistic. Indeed, 11.6 % and 11.3% of the markers in Cohorts 1 and 2, respectively, are significant at the 0.05 level with EM-HE, whereas 6.0% and 7.5% are significant with the NP statistic.

We used the linkage analysis on Cohort 1 to develop hypothesis that could be tested in Cohort 2. Two genomic regions were "replicated" in Cohort 2 using the same statistical method (D5S1456 and D11S2359). An additional two signals were found to be nominally significant by EM-HE in Cohort 1 and "replicated" with NP in Cohort 2. Three of these four signals remained nominally significant when extended pedigrees were analyzed.

## Figures and Tables

**Table 1:** Family resemblance for log AVGRES in cohort

**Relationship**	**N**	**Correlation**
Sister/Sister	97	0.293
Brother/Sister	181	0.216
Brother/Brother	90	0.227
Spouse	348	0.045

**Table 2:** Significant NP and EM-Haseman-Elston scores from Cohort 1 and the "replication" scores from Cohort 2

		**Cohort 1**		**Cohort 2**	
			
**Marker**	**Distance^A^**	**NP**	**EM-HE**	**NP**	**EM-HE**
D1S534	152	0.33	1.83	1.39	1.01
D1S1653	164	-0.04	1.87	1.98	1.47
D2S410	125	1.74	0.80	-0.32	-0.19
D2S1384	200	1.17	2.00	-0.89	0.44
D2S2944	210	1.12	1.88	-0.96	0.70
D4S2632	51	1.19	1.80	-0.16	0.20
D4S2394	130	1.71	0.57	-0.26	-0.35
D4S1625	146	1.71	0.46	0.13	-0.16
D5S1456	175	1.90	1.18	2.45	2.40
D9S1121	44	1.00	1.83	-0.14	1.16
GATA121A08	88	1.44	2.12	-0.44	-0.26
D10S1432	94	1.42	2.46	-0.45	0.15
D10S2327	101	1.36	2.14	0.64	1.00
D10S2470	113	0.33	1.74	0.74	-0.41
D10S1230	143	1.41	1.68	2.36	2.51
D11S2359	147	1.78	2.12	1.36	3.29
D14S742	12	1.67	3.00	0.22	-1.83
D14S1280	26	0.93	1.79	-0.03	-0.65
D14S608	28	0.98	1.89	0.00	-0.26
D14S297	32	0.86	2.14	0.03	-0.47
D14S53(A)	85	0.92	1.86	-0.55	-0.69
D14S606	92	1.65	2.75	-0.51	-0.09
GATA193A07	96	2.57	3.29	-0.80	-0.07
D14S610	97	2.58	3.30	-0.90	-0.13
D14S617	106	1.40	1.73	-1.73	-0.58
D14S1434	113	1.64	2.34	-1.31	-0.34
D16S2624	88	1.05	2.16	0.30	0.62
GATA178F11	3	2.89	4.19	0.63	0.69
D18S481	7	3.00	4.53	-0.05	0.01
D18S976	13	3.03	3.74	-0.71	-0.54
D18S843	28	2.36	3.16	-1.51	-1.70
D18S542	41	2.27	2.20	-1.07	-1.75
D18S877	54	2.02	3.21	-0.90	-1.47
D18S535	64	1.89	2.97	-0.82	-0.98
D19S1034	21	1.45	1.81	0.16	0.40
D19S589	88	1.54	2.26	-0.41	1.31
D19S254	101	1.18	2.06	-0.63	1.11
D20S470	39	0.82	2.22	2.23	1.57
D20S477	48	0.60	1.97	1.50	1.64
D20S478	54	0.80	1.86	1.10	1.42

^A^Distance is from p-arm telomere of the sex-average Marshfield map . Duplicate marker D14S53 was retained for analysis but deleted from this table.

**Table 3:** Linkage analysis of extended families for markers with significant evidence in Cohorts 1 and 2^A^

**Marker**	**Distance**	**NP**	**EM-HE**
D1S534	152	0.89	0.81
D1S1653	164	1.62	1.18
D1S1679	171	1.60	1.40
D5S1471	172	1.54	1.92
D5S1456	175	2.05	2.14
D5S211	183	2.54	1.86
D11S912	131	1.20	1.47
D11S2359	147	1.47	2.86
D20S604	33	0.78	0.60
D20S470	39	1.85	1.08
D20S477	48	1.91	1.60

^A^Multipoint scores for the closest proximal and distal markers (except for D11S2359, which has no telomeric neighbor) are included.
